# Cost-Utility of an Online Education Platform and Diabetes Personal Health Record: Analysis Over Ten Years

**DOI:** 10.1177/19322968211069172

**Published:** 2022-01-05

**Authors:** Scott G. Cunningham, Andrew Stoddart, Sarah H. Wild, Nicholas J. Conway, Alastair M. Gray, Deborah J. Wake

**Affiliations:** 1 School of Medicine, University of Dundee, Dundee, UK; 2 Edinburgh Clinical Trials Unit, Usher Institute, The University of Edinburgh, Edinburgh, UK; 3 Usher Institute, The University of Edinburgh, Edinburgh, UK; 4 Health Economics Research Centre, Department of Public Health, University of Oxford, Oxford, UK

**Keywords:** self-management, health care delivery, health economics, education, eHealth, medical informatics, information technology

## Abstract

**Background and Aims:**

My Diabetes My Way (MDMW) is Scotland’s interactive website and mobile app for people with diabetes and their caregivers. It contains multimedia resources for diabetes education and offers access to electronic personal health records. This study aims to assess the cost-utility of MDMW compared with routine diabetes care in people with type 2 diabetes who do not use insulin.

**Materials and Methods:**

Analysis used the United Kingdom Prospective Diabetes Study (UKPDS) Outcomes Model 2. Clinical parameters of MDMW users (n = 2576) were compared with a matched cohort of individuals receiving routine care alone (n = 11 628). Matching criteria: age, diabetes duration, sex, and socioeconomic status. Impact on life expectancy, quality-adjusted life years (QALYs), and costs of treatment and complications were simulated over ten years, including a 10% sensitivity analysis.

**Results:**

MDMW cohort: 1670 (64.8%) men; average age 64.3 years; duration of diabetes 5.5 years. 906 (35.2%) women: average age 61.6 years; duration 4.7 years. The cumulative mean QALY (95% CI) gain: 0.054 (0.044-0.062) years. Mean difference in cost: –£118.72 (–£150.16 to –£54.16) over ten years. Increasing MDMW costs (10%): –£50.49 (–£82.24-£14.14). Decreasing MDMW costs (10%): –£186.95 (–£218.53 to –£122.51).

**Conclusions:**

MDMW is “dominant” over usual care (cost-saving and life improving) in supporting self-management in people with type 2 diabetes not treated with insulin. Wider use may result in significant cost savings through delay or reduction of long-term complications and improved QALYs in Scotland and other countries. MDMW may be among the most cost-effective interventions currently available to support diabetes.

## Introduction

Global diabetes prevalence is increasing rapidly. The International Diabetes Federation predicts that the number of people with diabetes will rise by 51% worldwide to 700 million by 2045.^1^ In the United Kingdom, it is predicted that annual health service spending on diabetes will rise to £16.9 billion by 2035.^2^ There is therefore urgent need for low-cost, effective solutions to improve diabetes management to help prevent or delay complication and treatment costs.

Self-management solutions are becoming more prevalent, particularly those that use technology. More recent innovations combine mobile technologies with human coaching,^3-6^ while others aim to support education and shared decision-making by allowing individuals to access their electronic personal health record (ePHR).^7,8^ In addition to these services, many hundreds more are available as stand-alone apps that support the management of glucose, activity, carbohydrate intake, weight, blood pressure, medication, and many other topics relevant to diabetes.^9,10^

**Figure 1.**
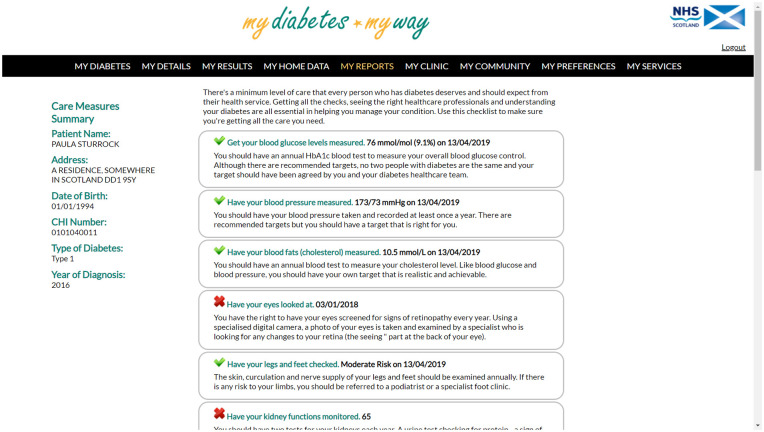
System screenshot from MDMW showing Care Measures Summary. Abbreviation: MDMW, My Diabetes My Way.

My Diabetes My Way (MDMW) is Scotland’s interactive website and mobile app for people with diabetes and their caregivers.^11^ The aim of MDMW is to support people with their self-management, by providing an accessible resource that encourages active participation in routine care. The website contains multimedia resources for diabetes education and offers people with diabetes access to their electronic personal health record (ePHR), facilitating personalized advice. The MDMW ePHR contains test results from general practice, secondary care systems, labs, and specialist screening systems, based on a clearly defined, patient-focused data set. These data are collated and linked through SCI-Diabetes, Scotland’s shared diabetes record^12^ before being made available to people with diabetes who have registered for records access through MDMW (see, for example, screen in Figure 1).

The aim of the MDMW ePHR is to enhance communications between people with diabetes and their health care teams, to encourage patient involvement, and to support shared decision-making. Previous analyses have demonstrated increased activity and usage trends,^13^ user-reported benefits,^14^ and improved changes in key metabolic parameters^15^ in users of MDMW compared with nonusers with similar clinical characteristics. Measuring cost-utility is essential to demonstrate further evidence of its value.

MDMW is referenced in the National Service Model for Home and Mobile Health Monitoring report^16^ as the service analyzed for the United4Health Telehealth Model for Diabetes in Scotland. This analysis demonstrated significant cost savings of €230 for €33 invested, by supporting better personal outcomes and experiences for citizens through supported self-management and online records access. Savings were derived predominantly from reduced acute admissions and through fewer primary, outpatient, and emergency department contacts. Following this publication, we aimed to extend our understanding of the service impact by identifying and applying a new robust and validated analysis model to the available data.

The United Kingdom Prospective Diabetes Study (UKPDS)^17^ was a randomized, multicenter trial that originally ran for 20 years from 1977 to 1997. This landmark study showed that the complications of type 2 diabetes can be reduced by improving blood glucose and blood pressure control. Based on the outcomes of the study, a computer simulation–based modeling tool (The UKPDS Outcomes Model2) was developed to estimate the impact of health interventions on people with type 2 diabetes.

The inputs to the model are individual patient-level data, including demographics, clinical risk factors, and complication history. These data are analyzed using risk equations for diabetes complications, including cardiovascular outcomes, amputation, blindness, renal failure, and mortality. The outputs then simulate estimated annual incidence of death or complications, including secondary events, life expectancy, and quality-adjusted life years (QALY).^18^ The UKPDS Outcomes Model2, the second iteration of the model, draws on additional data collected from UKPDS post-study follow-up to 2007 and follows American Diabetes Association guidance on developing diabetes decision models. This model has been internally validated over a 25-year time period^18^ and has been externally validated at international events such as the Mount Hood Challenge.^19^

The purpose of this study is to assess the cost-utility of a data-driven education platform and ePHR for diabetes, compared with routine diabetes care. It focuses on a clearly defined cohort of people with type 2 diabetes who are not prescribed insulin, consistent with the original UKPDS population.

## Materials and Methods

Data for this study were based on an anonymized, patient-level data extract from SCI-Diabetes, current and accurate as of July 2018. For the MDMW cohort, people with type 2 diabetes who have not been prescribed insulin and who had logged in to MDMW at least once since its launch in December 2010 were identified on SCI-Diabetes. This cohort was chosen as the largest subgroup of patients with type 2 diabetes in our population. Individuals were required to have received at least three years of routine follow-up since their first MDMW login to qualify for inclusion in the analysis. For the comparison cohort, nonusers on MDMW were identified on SCI-Diabetes with the following attributes: type 2 diabetes, not previously prescribed insulin, receiving routine care alone, not registered with MDMW, and alive on the index date for their matched MDMW user. Five nonusers were matched and analyzed for every MDMW user. Matching criteria were age (within two years), duration of diabetes (within one year), sex (exact match), and socioeconomic status, based on the Scottish Index of Multiple Deprivation (SIMD)^20^ quintile (exact match).

A license for the UKPDS Outcomes Model2^18^ was obtained and the necessary software was installed on a secure environment for the analysis. The software package includes a data input and validation spreadsheet, which is then loaded and processed by an executable package to generate outputs.

A mixture of summary and individual longitudinal test results were added to the UKPDS model. The latest clinical parameters that were available in SCI-Diabetes prior to first MDMW login (or matched date) were added to the summary worksheet for both cohorts, including hemoglobin A1c (HbA1c), systolic blood pressure, weight, and estimated glomerular filtration rate (eGFR). For tests that are not included in the SCI-Diabetes data set (hemoglobin, white blood cell count, and heart rate), fixed values within “normal” range were used for all participants. All available HbA1c results collected following first access were then added to the HbA1c worksheet. We aimed to also include systolic blood pressure in the same way, but these data were not available at the time of analysis, meaning only the baseline blood pressure was analyzed. Missing data were imputed by the modeling software before all clinical data added to the spreadsheet were validated by the model. A 3.5% time-related discount rate was applied to all future costs and effects in line with UK practice and the National Institute for Clinical Excellence (NICE) reference case. This is “an annual percentage by which future costs and/or health effects are reduced to reflect the diminishing value of results occurring in the future relative to those earned in the present.”^21^ No adjustments were made to the default UKPDS costs for the treatment of events (see Table 1). This shows the costs for treating complications: inpatient and outpatient visits, procedures, tests, general practitioner (GP) and practice nurse consultations, and so on.

**Table 1.** Complication Costs (2018) Applied to the UKPDS Model.

Male	Cost until age 50 years			Cost until age 60 years			Cost until age 70 years			Cost until age 80 years			Cost until age 90 years		
	At time of event		In subsequent years	At time of event		In subsequent years	At time of event		In subsequent years	At time of event		In subsequent years	At time of event		In subsequent years
	Fatal cost	Nonfatal cost	Cost	Fatal cost	Nonfatal cost	Cost	Fatal cost	Nonfatal cost	Cost	Fatal cost	Nonfatal cost	Cost	Fatal cost	Nonfatal cost	Cost
IHD	4153	9583	1423	4629	10 631	1869	5093	11 543	2449	5546	12 332	3163	5988	13 021	3987
MI	2062	6775	1397	2484	7339	1825	2908	7863	2378	3332	8358	3052	3754	8831	3820
Heart failure	0	3550	1935	0	4170	2446	0	4776	3091	0	5356	3854	0	5905	4698
Stroke	4629	6614	1461	5045	7840	1881	5460	9006	2438	5875	10 064	3140	6291	10 997	3971
Amputation	0	11 480	2827	0	12 245	3403	0	12 920	4091	0	13 523	4855	0	14 071	5651
Blindness		2665	967		3145	1191		3753	1501		4476	1913		5279	2428
Renal failure	0	19 190	19 190	0	19 190	19 190	0	19 190	19 190	0	19 190	19 190	0	19 190	19 190
Ulcer		6599	1000		6599	1000		6599	1000		6599	1000		6599	1000
Cost in absence of complications	771			991			1297			1708			2236		
Female	Cost/utilities until age 50 years			Cost/utilities until age 60 years			Cost/utilities until age 70 years			Cost/utilities until age 80 years			Cost/utilities until age 90 years		
	At time of event		In subsequent years	At time of event		In subsequent years	At time of event		In subsequent years	At time of event		In subsequent years	At time of event		In subsequent years
	Fatal cost	Nonfatal cost	Cost	Fatal cost	Nonfatal cost	Cost	Fatal cost	Nonfatal cost	Cost	Fatal cost	Nonfatal cost	Cost	Fatal cost	Nonfatal cost	Cost
IHD	4546	10 135	1710	4478	11 150	2200	5482	12 031	2829	5933	12 793	3590	6658	17 518	4448
MI	7464	12 218	1682	2288	7748	2153	3289	8265	2752	3713	8754	3468	2280	11 706	4266
Heart failure	0	3959	2244	0	4585	2804	0	5192	3496	0	5768	4298	0	5222	5165
Stroke	5010	7183	1737	4824	8409	2203	5841	9557	2810	6256	10 587	3562	7156	11 126	4434
Amputation	0	11 958	3166	0	12 699	3787	0	13 354	4512	0	13 942	5301	0	17 642	6108
Blindness		2966	1191		3492	1445		4146	1791		4908	2243		1830	2798
Renal failure	0	19 190	19 190	0	19 190	19 190	0	19 190	19 190	0	19 190	19 190	0	19 190	19 190
Ulcer		6599	1000		6599	1000		6599	1000		6599	1000		6599	1000
Cost in absence of complications	991			1239			1580			2034			2604		

All values given in pounds sterling.

Abbreviations: UKPDS, United Kingdom Prospective Diabetes Study; IHD, ischemic heart disease; MI, myocardial infarction.

Therapy costs input to the model were taken from those outlined in a recent cohort study based on Scottish data.^22^ This ensures that medication costs are accurately measured: that is, the costs of metformin, liraglutide, and so on. At the time of analysis, operating costs of MDMW are approximately £3.42 per registered user per annum and this figure was added to the costs of the MDMW group.

The model was set to run with 100 loops of 1000 bootstraps, with the mean value averaged for each individual patient to generate aggregated outputs. MDMW’s estimated impact on life expectancy, quality-adjusted life years (QALY), diabetes treatment costs, and anticipated diabetes-related complications costs were generated by the model over a ten-year time period, focusing on cardiovascular outcomes. We selected impact on outcomes over ten years to allow time for the model to generate a sufficient number of simulated complications.

To support a sensitivity analysis, and to demonstrate robustness of the results beyond cardiovascular complications alone, we modeled data for all complications along with four additional analyses. The first two applied an additional 10% therapy cost to the MDMW group for cardiovascular complications, and then to all complications. This was to account for any additional therapy costs that may arise through improved compliance with medication regimens. The final two analyses applied a 10% reduction to the MDMW therapy costs in the event of fewer face-to-face appointments and admissions as described in previous independent analysis.^16^ The input parameters for all six scenarios are shown in Table 2.

**Table 2.** Therapy Costs (2018) Applied to the UKPDS Model for Each Analysis.

	MDMW	Match
CVD complications—Equal therapy costs		
Therapy cost prior to complication	86.53	83.11
Number of years to apply from start	10	10
Therapy cost post complication	189.95	186.53
Number of years to apply from complication	10	10
All complications—Equal therapy costs		
Therapy cost prior to complication	86.53	83.11
Number of years to apply from start	10	10
Therapy cost post complication	189.95	186.53
Number of years to apply from complication	10	10
CVD complications—MDMW 10% increased therapy		
Therapy cost prior to complication	94.84	83.11
Number of years to apply from start	10	10
Therapy cost post complication	208.60	186.53
Number of years to apply from complication	10	10
All complications—MDMW 10% increased therapy		
Therapy cost prior to complication	94.84	83.11
Number of years to apply from start	10	10
Therapy cost post complication	208.60	186.53
Number of years to apply from complication	10	10
CVD complications—MDMW 10% reduced therapy		
Therapy cost prior to complication	78.22	83.11
Number of years to apply from start	10	10
Therapy cost post complication	171.30	186.53
Number of years to apply from complication	10	10
All complications—MDMW 10% reduced therapy		
Therapy cost prior to complication	78.22	83.11
Number of years to apply from start	10	10
Therapy cost post complication	171.30	186.53
Number of years to apply from complication	10	10

All values given in pounds sterling.

Abbreviation: UKPDS, United Kingdom Prospective Diabetes Study; CVD, cardiovascular disease; MDMW, My Diabetes My Way.

## Results

The MDMW cohort consisted of 2576 individuals, of whom 1670 (64.8%) were men, with an average age of 64.3 years and duration of diabetes of 5.5 years. Women within the MDMW cohort had an average age of 61.6 years and diabetes duration of 4.7 years. In the year after the index date, MDMW users’ mean HbA1c reduced from 56.6 mmol/mol (7.3%) to 54.9 mmol/mol (7.2%)—women; and from 56.9 mmol/mol (7.4%) to 55.1 mmol/mol (7.2%)—men. A breakdown of the cohort matching is shown in Table 3.

**Table 3.** Cohort Matching Comparison at Index Date.

Matching criteria	MDMW cohort		Matched cohort	
	Male	Female	Male	Female
Total subjects	1670 (64.8%)	906 (35.2%)	7534 (64.8%)	4092 (35.2%)
Age	64.3 years	61.6 years	64.5 years	61.9 years
Duration of diabetes	5.5 years	4.7 years	5.8 years	5.0 years
HbA1c at index date	56.9 mmol/mol (7.4%)	56.6 mmol/mol (7.3%)	56.3 mmol/mol (7.3%)	55.9 mmol/mol (7.3%)
Ethnicity				
White	1608 (96.3%)	888 (98.0%)	7006 (93.0%)	3776 (92.3%)
African Caribbean	9 (0.5%)	1 (0.1%)	52 (0.7%)	32 (0.8%)
Asian/Indian	53 (3.2%)	17 (1.9%)	477 (6.3%)	285 (7.0%)
Socioeconomic status—Scottish Index of Multiple Deprivation (SIMD)^20^				
SIMD 1 (least affluent)	235 (14.1%)	153 (16.9%)	1075 (14.3%)	699 (17.1%)
SIMD 2	269 (16.1%)	175 (19.3%)	1232 (16.4%)	753 (18.4%)
SIMD 3	312 (18.7%)	168 (18.5%)	1437 (19.1%)	809 (19.8%)
SIMD 4	390 (23.4%)	189 (20.9%)	1775 (23.6%)	880 (21.5%)
SIMD 5 (most affluent)	464 (27.8%)	221 (24.4%)	2016 (26.8%)	950 (23.2%)

Abbreviations: MDMW, My Diabetes My Way; SIMD, Scottish Index of Multiple Deprivation.

**Figure 2.**
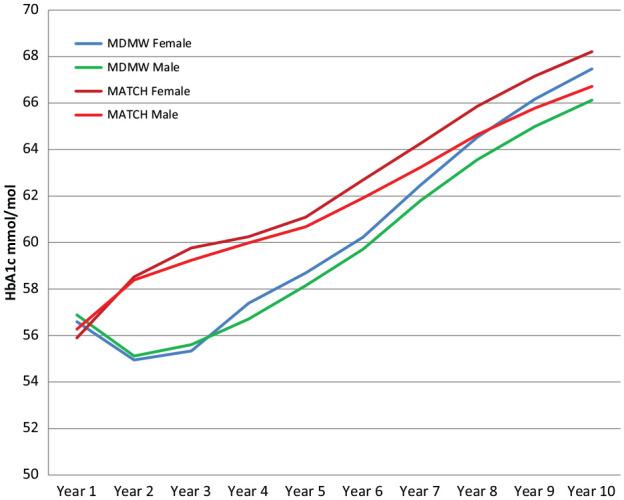
Comparison of actual and modeled mean HbA1c for MDMW users and nonusers by sex—Years 1 to 10. Abbreviations: HbA1c, hemoglobin A1c; MDMW, My Diabetes My Way.

The mean HbA1c within the matched cohort of similar individuals receiving routine care alone (n=11 626) increased from 55.9 mmol/mol (7.3%) to 58.5 mmol/mol (7.5%)—women; and from 56.3 mmol/mol (7.3%) to 58.4 mmol/mol (7.5%)—men, during the same period (see Figure 2). After three years of follow-up, mean HbA1c of MDMW users was between 4.4 mmol/mol (women) and 3.6 mmol/mol (men) below that of their matched counterparts after three years of follow-up (*P* < .001).

The model outputs for each analysis are shown in Table 4. The cumulative mean QALY (95% CI) gain for MDMW users was 0.054 (0.044-0.062) years. This is the equivalent of 19.71 (16.06-22.63) days. Over a ten-year period, MDMW users’ cardiovascular disease therapy costs increased by £28.30 (£27.30-£29.95), while complication costs showed a difference of –£147.03 (–£177.90 to –£83.40). This leads to a mean cost difference of –£118.72 (–£150.16 to –£54.16) over a ten-year period. At the time of analysis, operating costs of MDMW are approximately £3.42 per registered user per annum, equating to a return on investment of well over 3:1 during a ten-year period. Table 4 shows that all six scenarios modeled showed total cost savings, regardless of the costs and complications selected. In the subsequent cardiovascular-focused simulations, increasing MDMW costs by 10% results in a mean cost difference of –£50.49 (–£82.24 to £14.14). Decreasing MDMW costs by 10% results in a difference of –£186.95 (–£218.53 to –£122.51).

**Table 4.** UKPDS Model Outputs for Each Analysis. Life Expectancy in Years—Costs (2018) Provided in Pounds Sterling.

	Life expectancy	MCE	95% CI		Total QALE	MCE	95% CI		Therapy costs	MCE	95% CI		Cost of complications	MCE	95% CI		Total cost	MCE	95% CI	
			Lower	Upper			Lower	Upper			Lower	Upper			Lower	Upper			Lower	Upper
CVD complications—Equal therapy costs																				
All	7.5089	0.0018	7.4690	7.5833	5.9962	0.0014	5.9638	6.0571	685.0700	0.2097	680.4755	692.7390	14 582.7983	7.2658	14 417.6992	14 852.0095	15 267.8683	7.4022	15 100.4136	15 544.6128
MDMW—Group 1	7.5586	0.0041	7.5172	7.6301	6.0402	0.0033	6.0051	6.1004	708.2405	0.4911	703.6944	716.1989	14 462.4348	16.2849	14 319.7817	14 732.9726	15 170.6753	16.6102	15 026.0724	15 447.8457
Match—Group 2	7.4979	0.0020	7.4590	7.5728	5.9864	0.0016	5.9546	6.0482	679.9369	0.2316	675.2269	687.5566	14 609.4629	8.1089	14 441.6792	14 878.9351	15 289.3998	8.2593	15 119.8669	15 565.6846
Difference 1, 2	0.0608	0.0042	0.0472	0.0695	0.0538	0.0033	0.0440	0.0622	28.3036	0.5196	27.2985	29.9506	−147.0281	17.5055	−177.9045	−83.4036	−118.7245	17.8657	−150.1571	−54.1643
All complications—Equal therapy costs																				
All	7.5089	0.0018	7.4690	7.5833	5.9962	0.0014	5.9638	6.0571	705.2021	0.2265	700.1479	713.6431	14 582.7983	7.2658	14 417.6992	14 852.0095	15 288.0004	7.4209	15 121.1340	15 565.8058
MDMW—Group 1	7.5586	0.0041	7.5172	7.6301	6.0402	0.0033	6.0051	6.1004	728.2220	0.5307	722.9024	736.7515	14 462.4348	16.2849	14 319.7817	14 732.9726	15 190.6568	16.6514	15 043.0850	15 468.5422
Match—Group 2	7.4979	0.0020	7.4590	7.5728	5.9864	0.0016	5.9546	6.0482	700.1024	0.2502	695.0310	708.6259	14 609.4629	8.1089	14 441.6792	14 878.9351	15 309.5653	8.2803	15 140.7932	15 586.2101
Difference 1, 2	0.0608	0.0042	0.0472	0.0695	0.0538	0.0033	0.0440	0.0622	28.1197	0.5541	26.5212	29.3247	−147.0281	17.5055	−177.9045	−83.4036	−118.9085	17.9264	−151.1539	−54.3858
CVD complications—MDMW 10% increased therapy																				
All	7.5089	0.0018	7.4690	7.5833	5.9962	0.0014	5.9638	6.0571	697.4441	0.2156	692.7740	705.2527	14 582.7983	7.2658	14 417.6992	14 852.0095	15 280.2423	7.4045	15 112.7313	15 557.1189
MDMW—Group 1	7.5586	0.0041	7.5172	7.6301	6.0402	0.0033	6.0051	6.1004	776.4709	0.5391	771.4769	785.2146	14 462.4348	16.2849	14 319.7817	14 732.9726	15 238.9057	16.6426	15 094.0421	15 516.7315
Match—Group 2	7.4979	0.0020	7.4590	7.5728	5.9864	0.0016	5.9546	6.0482	679.9369	0.2316	675.2269	687.5566	14 609.4629	8.1089	14 441.6792	14 878.9351	15 289.3998	8.2593	15 119.8669	15 565.6846
Difference 1, 2	0.0608	0.0042	0.0472	0.0695	0.0538	0.0033	0.0440	0.0622	96.5340	0.5596	95.5509	98.5498	−147.0281	17.5055	−177.9045	−83.4036	−50.4941	17.8928	−82.2398	14.1370
All complications—MDMW 10% increased therapy																				
All	7.5089	0.0018	7.4690	7.5833	5.9962	0.0014	5.9638	6.0571	717.9385	0.2326	712.7810	726.5281	14 582.7983	7.2658	14 417.6992	14 852.0095	15 300.7367	7.4236	15 133.8105	15 578.6989
MDMW—Group 1	7.5586	0.0041	7.5172	7.6301	6.0402	0.0033	6.0051	6.1004	798.4502	0.5827	792.6082	807.8035	14 462.4348	16.2849	14 319.7817	14 732.9726	15 260.8850	16.6880	15 112.7555	15 539.7529
Match—Group 2	7.4979	0.0020	7.4590	7.5728	5.9864	0.0016	5.9546	6.0482	700.1024	0.2502	695.0310	708.6259	14 609.4629	8.1089	14 441.6792	14 878.9351	15 309.5653	8.2803	15 140.7932	15 586.2101
Difference 1, 2	0.0608	0.0042	0.0472	0.0695	0.0538	0.0033	0.0440	0.0622	98.3478	0.5965	96.7489	99.9093	−147.0281	17.5055	−177.9045	−83.4036	−48.6803	17.9585	−81.1956	15.8332
CVD complications—MDMW 10% reduced therapy																				
All	7.5089	0.0018	7.4690	7.5833	5.9962	0.0014	5.9638	6.0571	672.6959	0.2064	668.1702	680.2253	14 582.7983	7.2658	14 417.6992	14 852.0095	15 255.4942	7.4000	15 088.0960	15 532.0928
MDMW—Group 1	7.5586	0.0041	7.5172	7.6301	6.0402	0.0033	6.0051	6.1004	640.0101	0.4431	635.9119	647.1913	14 462.4348	16.2849	14 319.7817	14 732.9726	15 102.4449	16.5778	14 958.1028	15 378.9599
Match—Group 2	7.4979	0.0020	7.4590	7.5728	5.9864	0.0016	5.9546	6.0482	679.9369	0.2316	675.2269	687.5566	14 609.4629	8.1089	14 441.6792	14 878.9351	15 289.3998	8.2593	15 119.8669	15 565.6846
Difference 1, 2	0.0608	0.0042	0.0472	0.0695	0.0538	0.0033	0.0440	0.0622	−39.9268	0.4804	−41.1268	−38.3664	−147.0281	17.5055	−177.9045	−83.4036	−186.9549	17.8385	−218.5255	−122.5077
All complications—MDMW 10% reduced therapy																				
All	7.5089	0.0018	7.4690	7.5833	5.9962	0.0014	5.9638	6.0571	692.4657	0.2228	687.5148	700.7613	14 582.7983	7.2658	14 417.6992	14 852.0095	15 275.2640	7.4184	15 108.4575	15 552.9127
MDMW—Group 1	7.5586	0.0041	7.5172	7.6301	6.0402	0.0033	6.0051	6.1004	657.9939	0.4787	653.1967	665.6994	14 462.4348	16.2849	14 319.7817	14 732.9726	15 120.4287	16.6149	14 973.4144	15 397.3315
Match—Group 2	7.4979	0.0020	7.4590	7.5728	5.9864	0.0016	5.9546	6.0482	700.1024	0.2502	695.0310	708.6259	14 609.4629	8.1089	14 441.6792	14 878.9351	15 309.5653	8.2803	15 140.7932	15 586.2101
Difference 1, 2	0.0608	0.0042	0.0472	0.0695	0.0538	0.0033	0.0440	0.0622	−42.1085	0.5124	−43.9499	−40.8999	−147.0281	17.5055	−177.9045	−83.4036	−189.1366	17.8945	−221.2088	−124.7293

Life expectancy in years—Costs (2018) provided in pounds sterling.

Abbreviations: UKPDS, United Kingdom Prospective Diabetes Study; MDMW, My Diabetes My Way; MCE,Monte Carlo Error; CI, confidence interval; QALE = quality-adjusted life expectancy.

Table 5 shows a breakdown of the simulated complications in both cohorts, detailing the event rates over 10 years/10 000 population. The reference event costs used are “Male Cost Until Age 70” as shown in Table 1 and as provided by default in the modeling software. In addition to the baseline costs shown, the value to people with diabetes and the health service can be demonstrated in the reduction in all types of complication calculated by the model. These simulations include 59 fewer myocardial infarctions and 42 fewer strokes per 10 000 population over ten years.

**Table 5.** Estimated Number of Events and the Costs (2018) of Nonfatal Events/10 000 Population (Reference Event Costs: Male Cost Until Age 70 years—See Table 1).

	IHD at	£11 543	MI at	£7863	Heart failure at	£4776	Stroke at	£9006	Amputation at	£12 920	Blindness at	£3753	Ulcer at	£6599
	events	Total cost	events	Total cost	events	Total cost	Events	Total cost	Events	Total cost	Events	Total cost	Events	Total cost
MDMW cohort	701.941	£8 102 504.89	1068.94	£8 405 107.45	546.47	£2 609 928.26	609.28	£5 487 157.22	193.75	£2 503 250.00	327.52	£1 229 194.91	244.95	£1 616 447.59
Matched cohort	719.350	£8 303 455.26	1127.90	£8 868 663.36	604.77	£2 888 395.67	651.42	£5 866 679.41	207.77	£2 684 333.33	335.11	£1 257 681.04	251.18	£1 657 524.88
Difference	–17.409	–£200 950.37	–58.95	–£463 555.91	–58.31	–£278 467.40	–42.14	–£379 522.19	–14.02	–£181 083.33	–7.59	–£28 486.12	–6.22	–£41 077.29

Abbreviation: IHD, ischemic heart disease; MI, myocardial infarction; MDMW, My Diabetes My Way.

**Figure 3.**
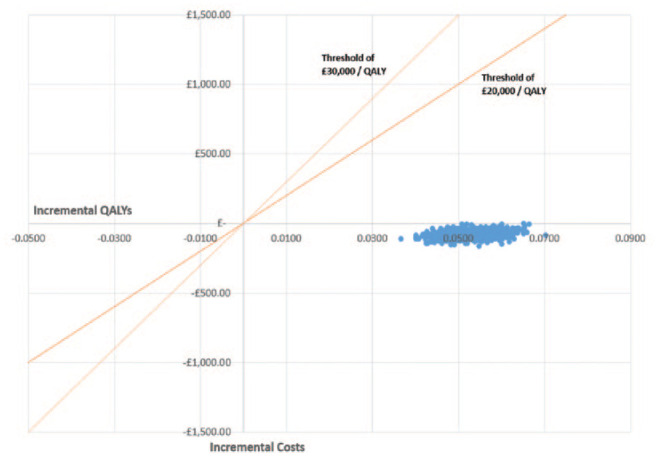
Scatter plot of incremental costs vs incremental QALYs for MDMW vs usual care. Abbreviations: QALYs, quality-adjusted life years; MDMW, My Diabetes My Way.

Figure 3 shows a scatterplot of the difference in costs and QALYs (MDMW minus control) from the 1000 bootstrapped simulations generated by the model. As per standard NICE reference case methodology,^23^ interventions are typically compared by comparing their incremental cost per QALY to a “threshold” of £20 000 to £30 000 per QALY. The proportion of simulated estimates that fall below the threshold line are interpreted as the probability that the intervention is cost-effective relative to its comparator, the rationale being that any additional funds spent to implement it couldn’t be used somewhere else in the system to produce more health (measured here in QALYs). As Figure 3 shows, all our simulations were below the 20k and 30k lines, and even indicated long-term cost savings as well as health improvements, a situation referred to as a “dominant” option in economic theory.

## Limitations of Study

This analysis focuses predominantly on changes in HbA1c although several baseline clinical results were used to populate the UKPDS summary worksheet. For a more robust future analysis, historical systolic blood pressure will also be included as this was a key indicator identified in the UKPDS trial, meaning that the results presented in this article may be conservative.

The analysis uses standard UKPDS Outcomes Model bootstraps, which deal with uncertainty in the model parameters by repeatedly re-estimating all equations from the underlying data set using bootstrapped resamples. It does not, however, deal with other uncertainties, for example, in therapy and treatment costs, quality of life, and so on.

MDMW was initially accessed in its first few years by small numbers of self-selecting and potentially more motivated individuals. However, we believe this effect has reduced in recent years following increasing MDMW usage among the overall diabetes population, as published in the annual Scottish Diabetes Survey.^24,25^ Previously, lack of awareness of the intervention was the biggest barrier to use.^15^ The demography of registrants is now increasingly similar to the background population, although still slightly younger, on average.^13^ Use by more motivated individuals will not necessarily result in a positive impact on evaluation as they may tend to have better baseline metabolic values with limited opportunity for significant change.

In this study, we have matched MDMW users with nonusers for sociodemographic and other parameters described earlier. However, as shown in Table 3, there are minor differences in some demographic characteristics. The MDMW cohort is marginally younger, with a slightly shorter duration of diabetes. The breakdown of ethnicity also shows that the MDMW cohort contains a higher proportion of individuals in a “white” ethnic group, which may affect the likelihood of developing complications, however, the UKPDS model adjusts for this.

Matched cohort analyses cannot account for factors such as personality and motivation to make positive lifestyle choices using the data and guidance provided by the system. However, review of the longitudinal HbA1c results (actual and imputed by the UKPDS model) demonstrate very similar HbA1c in the pre-intervention period between cohorts, with a significant rapid temporal change in line with adoption of MDMW in both males and females, suggesting a clear intervention effect that may wane with time should the progression of diabetes follow the pathway modeled in the UKPDS Outcomes Model2—see Figure 2. That said, the sizable benefits obtained in the short term would be very hard to offset with the relatively negligible running costs even if the program were to run for several years on each patient after the benefits had reduced. Limitations of the UKPDS Outcomes Model2 are documented elsewhere^18^ and it should also be noted that it was originally created in a different era of diabetes management, but it is still considered a gold standard that has been incrementally updated over time.

This analysis describes the outputs using the data of patients with type 2 diabetes who are not on insulin. This provides a focus on the largest subgroup of patients with type 2 diabetes in our population, who are generally managed in primary care. Patients using insulin (type 1 and type 2) tend to be managed in secondary care in Scotland with different characteristics, requirements, and MDMW feature use, such as support for blood glucose monitoring, and so on. The role of MDMW within these patient cohorts will be analyzed in a separate study, using a more appropriate model for people with type 1 diabetes, given that UKPDS is currently only validated for type 2 diabetes.

## Discussion

This analysis suggests that MDMW is well below accepted UK thresholds for cost-effectiveness,^23^ and appears to have a high probability of being both cost-saving and life improving (albeit clinically insignificant) relative to usual care, a situation referred to as a “dominant” intervention in Health Economic terminology. In Scotland, there are currently 235 769 (88.1% of total type 2 diabetes population) people with type 2 diabetes not on insulin.^25^ Extrapolating these results, we can speculate that a potential ten-year cost saving could be approximately £28m (235 769 × £118.72) if every person in this category were to use the system and had similar benefits. Even with only 25% uptake of the service and similar benefits, there remains a potential saving of around £7m.

Extrapolating further to National Health Service (NHS) England, this impact may be considerably higher due to the larger diabetes population. There are currently approximately 2 839 074 people with type 2 diabetes not on insulin living in England (88.1% of 3 222 559 people with type 2 diabetes^26^). In this environment, the potential ten-year cost saving from events avoided and treatment costs could be as much as £337.1m (2 839 074 × £118.72) if every person in this category were to use the system. Again, with only 25% uptake of the service, considerable potential savings remain at £84.2m. We acknowledge that these extrapolations are based on a number of assumptions that would need to be validated to increase confidence in their reliability.

As described in the “Limitations” section of this article, we cannot rule out self-selection bias in the MDMW cohort. As the service scales up and engages a wider demographic, impact may increase due to a poorer baseline, or reduce due to lower motivation levels in the cohort. However, given the lower confidence interval on cost-difference was –£54.16, MDMW would remain cost saving.

This study suggests a cost saving impact, whereas other digital interventions cannot, due to the combination of effect on HbA1c and a very low cost of implementation and delivery. This is due to the automated nature of the product where data are collected using electronic systems and records linkage, with little reliance on human resource and face-to-face or virtual/telephone coaching, and so on. MDMW is available for as long as the person with diabetes wishes to engage with it. MDMW has also been designed to be scalable at very little additional annual cost and has the potential to span large geographical areas once commissioned. The reason for this is that it is based on a fixed annual funding cost, with no additional cost as new users register. Based on the 2018-2019 annual service cost for NHS Scotland, and the potential cost saving per person of £118.72 over ten years, MDMW is at least cost neutral when approximately 12 000 people are using it. In 2021, there are now more than 55 000 people in Scotland registered for the service with more than 30 000 active users, meaning cost of delivery is reduced to around £2.87 per registrant.

While cost utility is the main focus of this article, in practice it is often affordability and system impact that influence uptake of a product in the NHS. While My Diabetes My Way should be seen as a service to support self-management and complement and augment existing care and treatment, we present Table 6 as a comparison of acquisition costs alongside other established diabetes treatments and interventions. This outlines annual costs per patient, based on the costs of supplying the medications or interventions for a full calendar year. These are mean costs per patient of the direct intervention costs only (ie, excluding any changes in wider NHS resource use, such as fewer hospital admissions). These data are presented instead of a formal return on investment due to the incompleteness of available data for comparison in the public domain. In practice, the effect of MDMW would be to enhance the effectiveness of current standard of care and the list of interventions in Table 6 would be offered to those who would be suitable as needed.

**Table 6.** Comparison of Annual Costs (2018) of Established Diabetes Interventions.

Intervention	Cost/year	Notes
Continuous glucose monitor—Dexcom	£3911.94	$4720—Sensors $349 for 30 days + Transmitters $475^27^
Flash continuous glucose monitor—Libre	£1350	£50 reader + £50 sensor × 26^28^
Liraglutide	£951.27	1.2 mg once daily^29^
Insulin pump	£500	~£2000 but should last 4 years^30^
Acarbose	£139.65	150 mg/day^29^
Diabetes education and self-management for ongoing and newly diagnosed (DESMOND)	£76.00	Per participant^10^
X-PERT	£65.00	Per participant^10^
Metformin	£38.61	1.5 g/day^29^
My Diabetes My Way	£3.42	

Comparison of mean HbA1c differences between the MDMW and matched cohort show improvements in these results in the initial three-year period. Most of these HbA1c data are derived from observed data and, after this point, the majority of HbA1c data is simulated by the model, with the subsequent time point varying based on the index date. It is expected therefore that the model essentially assumes that these data converge over a longer time period.

Previous studies have shown that even modest improvements in glycemic control can reduce rates of complication in diabetes.^31^ This analysis is based on a service that has a ten-year track record. It uses real-world, routinely collected clinical data, as opposed to data collected in a small-scale trial environment, which may not accurately predict genuine benefits in a live population.

A recent systematic review indicated that people with diabetes and health care services could benefit from using high-value services.^32^ We have been unable to find evidence of another diabetes intervention demonstrating such a return on investment as MDMW. Even Metformin, hailed as a cheap and effective therapeutic option, shows only a modest cost saving.^33^ It would be expected that, when a new treatment is significantly more effective and less costly than usual care, it should be widely adopted.

The MDMW product has evolved significantly ever since data collection was completed for this analysis, with the addition of eight Quality Institute for Self-Management Education and Training (QISMET)-accredited online structured education courses, the release of a mobile app, remote communication tools (with health care professionals), and remote data upload (eg, activity and glucose tracking). This has been achieved with no additional running cost and we expect this functionality to add further value for users of this service.

The next steps for MDMW will include an extension of the aforementioned analysis to include additional continuous data, including blood pressure, which played a significant role in risk reduction during the UKPDS trial, along with cholesterol, weight, and so on. This may mean that the results presented in the foregoing are an underestimate of the actual savings that may be realized by MDMW. We are independently assessing the impact on service delivery efficiency and care processes, which will be relevant to the NHS systems that adopt it. We also aim to use the UKPDS model to assess the data of people with type 2 diabetes who are prescribed insulin and determine an appropriate model to analyze the data of those with type 1 diabetes.

## Conclusions

The result of this analysis indicates that use of MDMW is expected to be “dominant” over usual care (both cost saving and life improving), in supporting self-management in people with type 2 diabetes not treated with insulin. Operating costs at the time of analysis were approximately £3.42 per registered diabetes patient per annum. This equates to a potential return on investment of well over 3:1 during a ten-year period based on the results shown by the UKPDS outcomes model. Wider use of the MDMW service could result in significant cost savings through delay or reduction of long-term complications and increased life expectancy in health care systems where it is used and adopted. This study demonstrates for the first time the potential of a low-cost, scalable digital intervention to deliver population-based benefits and cost savings.
